# On artherogenic index of plasma in sickle cell anaemia patients

**DOI:** 10.11604/pamj.2019.32.141.17166

**Published:** 2019-03-25

**Authors:** Akinsegun Abduljaleel Akinbami, Ebele Ifeyinwa Uche, Aishatu Maude Suleiman, Ann Abiola Ogbenna, Festus Olusola Olowoselu, Benjamin Augustine, Mulikat Adesola Badiru, Rafat Abiodun Bamiro, Omolara Risqat Kamson

**Affiliations:** 1 Department of Haematology and Blood Transfusion, Lagos State University, College of Medicine PMB 21266, Ikeja, Lagos, Lagos State, Nigeria; 2 Department of Haematology and Blood Transfusion, Faculty of Basic Clinical Sciences, College of Health Sciences, Ahmadu Bello University, Zaria; 3 Department of Haematology and Blood Transfusion, Faculty of Clinical Sciences, College of Medicine, University of Lagos, Nigeria; 4 Department of Hematology and Blood Transfusion, Lagos State University, Teaching Hospital, PMB 21266, Ikeja, Lagos State, Nigeria

**Keywords:** Artherogenic index of plasma, lipid profile, sickle cell disease

## Abstract

**Introduction:**

Sickle cell anaemia (SCA) is an inherited abnormality of haemoglobin associated with reduced life expectancy. Patients' complications include dyslipideamia. This study was aimed at determining the artherogenic index of plasma (AIP) in sickle cell anaemia patients and compares the value to HbAA controls value. A high AIP is strongly predictive of elevated cardiovascular risk.

**Methods:**

A comparative study was conducted among SCA patients attending the haematology clinic, Lagos State University Teaching Hospital (LASUTH) and HbAA Phenotype controls. A total of 304 participants were recruited consisting of equal numbers of SCA and HbAA controls. Single lipid profiles were done; logarithms of triglycerides/high density lipoprotein were calculated to obtain AIP and lipid profile ratios established for all participants.

**Results:**

There were lower mean values of Total Cholesterol (TC), High Density Lipoprotein(HDL) and Low Density Lipoprotein (LDL) amongst SCD participants than controls and higher mean values of triglycerides (TG) and Very Low Density Lipoprotein (VLDL) in SCD p < 0.05. The AIP in SCD ranges from -0.62 to 1.32 while that of controls ranges from -0.56 to 0.61.The mean AIP were 0.14 ± 0.29 and -0.009 ± 0.26 in SCD and controls respectively. P value = 0.002.

**Conclusion:**

AIP value is higher in sickle cell anaemia than controls, the former have lower mean values of TC, HDL and LDL and higher mean values of TG and VLDL.

## Introduction

In 1910 Dr James Herrick first described SCA in a Dental student in Chicago, USA [1]. Sickle cell gene is characterized by a point mutation in the 6^th^ codon of the haemoglobin gene in which adenine is replaced by thymine (GAG→GTG).The mutation results in the replacement of glutamic acid by valine on the 6^th^ amino acid in the β globin chain of the haemoglobin molecule. Considerable variation in clinical severity occurs in SCA patients despite possessing the same basic identical genetic mutation (GAG→GTG) [2]. Recognized causes of these variations include co-inheritance of α-thalassaemia [3], expression of adhesion molecules on white blood cells [4], steady state neutrophil counts and function [5], haemoglobin haplotypes and HbF concentrations [6], levels of transferrin/C-reactive protein [7], socio-economic status [8], plasma level of IgG and in particular IgG3 [9], levels of circulating immune complexes [10] and dyslipidaemia [11].

**Lipid Profile:** Dyslipidaemia depicts deranged plasma concentration of the lipid profile [12]. Lipid profile consists of total cholesterol (TC), high-density lipoprotein (HDL)-cholesterol, triglycerides (TG), calculated very low density lipoprotein (VLDL) -cholesterol and low density lipoprotein (LDL)-cholesterol. Lipid profiles are useful tests in determining cardiovascular risk and stroke resulting from occlusion of the micro-vasculature and venous thrombo-embolism (VTE) [13] in SCA and general population. These complications are common causes of morbidity in SCA [11]. Pulmonary embolism and hypertension are also common causes of morbidity and mortality in SCA consequent upon thrombo-embolic disease as a result of dyslipidaemia [14].

**Dyslipidaemia, cardiovascular diseases and venous thrombo-embolism in SCA:** There are several reports on risk factors of cardiovascular diseases (CVD), these are dyslipidaemia, smoking, poor diet, hypertension, sedentary life style and obesity [15-18]. SCA patients are known to be more predisposed to VTE than the general population [19]. Various reported processes involved in the development of VTE in SCA include dyslipidaemia and associated erythrocyte adhesions [20], platelet aggregation [21], coagulation defects [22], free hemoglobin-induced oxidative damage [23], leukocyte activation in the setting of chronic inflammation and erythrocyte-induced endothelial dysfunction [24]. However, amongst these risk factors, dyslipideamia is the most important [25]. In 2002 [26] LDL-C was used to predict first cardiovascular events in a study involving 27,939 apparently healthy American women, however, the prediction failed in 46% of those who had normal LDL-C levels. In 2015, a hospital based study involving 738 coronary heart disease and 157 control participants amongst Chinese Han population [27], it was reported by the authors that serum lipid ratios such as low-density lipoprotein-cholesterol (LDL-C)/HDL-C, triglycerides (TG)/HDL-C and total cholesterol/high-density lipoprotein-cholesterol (TC/HDL-C) were superior predictors of CVD than the single conventional lipid parameters. Logarithm of the ratio of TG and HDL-C known as artherogenic index of plasma (AIP) was first described in 2001 [28], but in 2017, it was considered to be stronger than the serum lipid ratios [29] because it has a stronger sensitivity that reflects the interactions between artherogenic and protective lipoprotein than the serum lipid ratios [30]. AIP categorizes CVD risks into low, intermediate and high risks based on values of <0.11, 0.11-0.21 and >0.21 respectively [31]. This study was aimed at establishing lipid profile reference ranges in SCA patients and HbAA general population and to compare artherogenic index of plasma (AIP) in both groups with a view to determining whether there is a higher AIP in SCA than the non-SCA patients, which could account for their more predisposition to cardiovascular diseases.

## Methods

**Study area:** The adult Haematology clinic LASUTH, the general outpatient department and blood donor clinics of the hospital were used for the study. The hospital was established as a cottage hospital in 1955 and in July 2001 it became a teaching hospital. It serves the Lagos metropolis with a population of approximately 20 million spread unevenly over 20 local government areas.

**Study population:** Adult SCA patients of Haematology clinic and HbAA volunteer participants attending general outpatients and blood donor clinics.

**Study design:** This was a comparative study of consenting adult SCA patients attending the Haematology clinic, LASUTH and consenting clients of general outpatient department and blood donor clinics who have HbAA Phenotype.

**Study period:** This study was done over a three months' period from June to August, 2018.

**Study site:** The laboratory of LASUTH/APIN (APIN Initiative of Nigeria) project located within the haematology clinic was used for the lipid profile assay, while haemoglobin electrophoresis of all participants were done at the haematology main laboratory.

**Sampling technique:** Steady state SCA patients as defined by Ballas SK [**32**] attending Haematology clinic LASUTH as well as consenting blood donors and general outpatient patients of LASUTH were recruited consecutively into the study. Only consenting participants who have HbAA phenotype and who met the inclusion criteria were used as control population. Heamoglobin phenotype of all cases and controls were performed using alkaline haemoglobin electrophoresis method at pH 8.4 before the lipid profile assays were done.

**Subjects/participants:** 1) Adults who are HbSS phenotype attending LASUTH Haematology clinic. 2) General outpatient and blood donor clients of LASUTH who have HbAA. 3) Phenotype served as controls.

### Inclusion criteria

**Adult HbSS phenotype patients:** 1) Alkaline Haemoglobin electrophoresis showing HbSS phenotype. 2) Age 18 years and above.

**General outpatient and donor clinic clients:** 1) Alkaline Haemoglobin electrophoresis showing HbAA phenotype. 2) Age 18 years and above.

### Exclusion criteria

**Adult HbSS phenotype patients:** 1) Non-consenting HbSS patients. 2) Other Hb phenotypes (e.g. HbSC, SD, Etc). 3) Non-fasting participants. 4) HbSS patients on lipid lowering medications. 5) HbSS patients who are hypertensive or diabetics.

**General outpatient and donor clinic clients:** 1) Non-consenting participants. 2) Other Hb phenotypes (e.g. AS, SC, AC etc). 3) Non fasting participants. 4) HbAA controls on lipid lowering medications. 5) HbAA controls who are hypertensive or diabetics.

**Sample size determination:** Sample size was determined using the statistical formula that applies to comparative studies [33].

Sample Size

$$n=\frac{z^{2}[P_{1}(100-P_{1})+P_{2}(100-P_{2}]}{d^{2}}$$

d2 Where: n = Sample Size; Z = 1.96 (at 95% confidence level); P_2_ = Reported Prevalence in general population = 1.3% [34]. P_1_ = Reported Prevalence in high risk population = 8.4% [35]. d = 5% (precision): n = 1.96^2^ [1.3(100 - 1.3) + 8.4(100 - 8.4)] / 5^2^ n = 3.8416 [1.3 x 98.7 + 8.4 x 91.6] / 25; n = 3.8416 [ 128.31 + 769.44] / 25; n = 3.8416 × 897.75 = 3488.79 = 137.95.9 25 25 n ~ 138 for each group of participants With an estimated non-response rate of 10%, attrition factor [36] = 100/100 - x. Attrition factor = 100 / 100 - 10 = 100 / 90 = 1.11. Total sample size for each group of participants with consideration for non response = 138 x 1.11 = 152. ~ 152.

**Ethical considerations and clearance:** Ethical approval was obtained from the Health Research Ethics committee of LASUTH Reference Number: LREC./06/10/1016. Ethical standards and procedures of the committee for human experimentation were adequately followed.

**Participant's informed consent:** The participants were informed about the study, as well as their rights and benefits. A written informed consent was obtained by means of voluntarily signed consent form. No participant was coerced in any way to participate in this study, which was at no cost to them.

**Confidentiality:** The names and initials of all participants were not used to guarantee confidentiality. Participants were assigned unique identification numbers. Paper records were stored in a cabinet in a secured room. Electronic data were password protected.

**Questionnaire administration and history taking:** With the use of an interviewer- administered questionnaire, each participant was interviewed to obtain relevant demographic and clinical data. Some of the questions asked in the questionnaire included age of diagnosis of sickle cell anaemia, history of blood transfusion, frequency of crisis per year, time of last acute painful crisis, history of last hospital admission, drug history and most frequent type of crisis to determine steady state status of the HbSS participants and history of lipid lowering drugs in both HbSS and controls to exclude those on this drug.

**Specimen collection:** From an intravenous access, under aseptic conditions using a vacutainer needle, 5mls of blood was collected into a lithium heparin vacutainer bottle for lipid profile of all participants. Another 2mls of blood sample was collected into a vacutainer EDTA bottle from all participants and properly mixed with the anticoagulant for alkaline Hb electrophoretic analysis in all participants to ensure the participants are either sickle cell anaemia patients or HbAA participants before the lipid profile assay.

**Lipid profile assay and calculation of atherogenic index of plasma and other ratios:** TC, TG and HDL assays were done with the cobas C111 chemistry auto-analyzer (Roche Diagnostic, Germany) which uses enzymatic, colorimetric method. VLDL and LDL-C were calculated from Friedewald formula [37]. VLDL as TG/2.2 (mmols/L) while LDL was calculated using LDL-C (mmols/L) = TC-VLDL-HDL. AIP was calculated in all participants [28] as log (TG/HDL-C) from the data generated, the lipid ratios such as low-density lipoprotein-cholesterol (LDL-C)/HDL-C, triglycerides (TG)/HDL-C and total cholesterol/high-density lipoprotein-cholesterol (TC/HDL-C) were also calculated from single lipid profile parameters assayed.

**Statistical analysis:** Data were analyzed by IBM SPSS (Statistical Package for Social Sciences, Inc.) statistics for windows version 20.0 Armonk, New York, USA. The continuous variables were presented as means ± standard deviation (SD). The Pearson chi squared tested for association between discrete variables. Independent t-test and analysis of variance (ANOVA) were used between the two groups. P value was considered to be statistically significant when ≤ 0.05.

## Results

A total of 304 participants were finally recruited after excluding non- consenting SCD patients and controls including 25 controls that have sickle cell trait and are double heterozygotes like HbSC, participants consisted of equal numbers of sickle cell patients and controls. The mean age of SCD was lower compared with controls and expectedly the mean BMI of SCD was also lower than that of controls. There are more males than females in both groups. The mean number of crisis/year in SCD was 1.6 ± 0.6 and the mean number of blood transfusion per year was 1.79 ± 1.28 (Table 1). The reference ranges of total cholesterol, triglyceride, HDL, VLDL and LDL in SCD and controls are presented in Table 2. The means of total cholesterol, triglyceride, HDL, VLDL and LDL in SCD and that of controls are shown in Table 3. Analysis of variance (ANOVA) of these means produced F ratio of 581.70 and 685.90 for SCD and controls respectively with p values of both groups being < 0.05. The ratio of LDL/HDL, TG/HDL and TC/HDL for both the SCD and controls are also presented in Table 3. ANOVA of the ratios also produced F ratio of 74.41 and 265.50 of both the SCD and controls respectively and p values < 0.05 for both groups. The artherogenic index of plasma (AIP) in SCD ranges from -0.62 to 1.32 while that of controls ranges from -0.56 to 0.61. The mean AIP were 0.14 ± 0.29 and -0.009 ± 0.26 in SCD and controls respectively (Table III). Using independent t-test for comparisons of SCD and controls, the AIP, MI, age, total cholesterol, high density lipoprotein and low density lipoprotein were all statistically significant with p values of < 0.05,while triglyceride and very low density lipoprotein were not statistically significant, p values were 0.76 and 0.64 respectively (Table 4). Similarly, the ratio of TG/HDL and TC/HDL were also statistically significant with a p value < 0.05, while the ratio of LDL/HDL did not reach a significant value.

**Table 1:** Participants’ age, BMI and gender

Parameters	SCA; N=152	Controls; N=152
Age (Years)	22±8.40	30.34±7.80
BMI	20.85±3.33	24.29±4.80
Sex: (M:F)	60:40	90:10

Key: BMI= Body mass index, SCA= Sickle Cell Anaemia, M:F = Male: Female

**Table 2:** Reference ranges of lipid profiles of SCA and controls

Parameters	SCA (mmols/L)	Controls (mmols/L)
TC	1.62-6.50	1.63-6.54
TG	0.53-4.20	0.40-4.94
HDL	0.09-2.19	0.59-2.25
VLDL	0.24-1.90	0.18-2.24
LDL	0.31-4.10	0.31-4.43

Key: SCA=Sickle cell anaemia; TC= Total cholesterol; TG= Triglyceride; HDL= High Density Lipoprotein; VLDL= Very Low Density Lipoprotein; LDL= Low Density Lipoprotein

**Table 3:** Mean values of lipid profiles in SCA and controls

Parameters	SCA (mmols/L)	Controls (mmols/L)
TC	3.29±0.75	4.03±0.91
TG	1.31±0.59	1.28±0.76
HDL	0.93±0.31	1.18±0.30
VLDL	0.59±0.26	0.57±0.33
LDL	1.75±0.61	2.27±0.71
LDL/HDL	2.25±2.08	2.01±0.77
TG/HDL	1.90±2.66	1.17±0.77
TC/HDL	4.13±3.22	1.17±0.77
Log TG/HDL (AIP)	0.14±0.29	-0.001±0.26

Key: SCA= Sickle Cell Anaemia, TC= Total Cholesterol, TG= Triglyceride, HDL= High Density Lipoprotein, VLDL= Very Low Density Lipoprotein, LDL= Low Density Lipoprotein; AIP= Artherogenic Index of Plasma

**Table 4:** Bivariate analysis of the parameters using independent t-test

Parameters	T-statistics	p-Value
Age	6.56	0.001
BMI	5.88	0.001
TC	6.19	0.001
TG	0.303	0.76
HDL	5.76	0.001
VLDL	0.468	0.64
LDL	5.63	0.001
TG/HDL	2.64	0.008
LDL/HDL	1.07	0.28
TC/HDL	8.92	0.001
Log TG/HDL (AIP)	3.84	0.002

Key: BMI= Body mass index, TC= Total Cholesterol, TG= Triglyceride, HDL= High Density Lipoprotein, VLDL= Very Low Density Lipoprotein, LDL= Low Density Lipoprotein.AIP= Artherogenic Index of Plasma

## Discussion

This study reported lower mean values of TC, HDL and LDL amongst Nigerian SCA adult participants than HbAA controls and higher mean values of TG and VLDL (p < 0.05). Similar finding was also reported among Nigerian SCA Children and adolescent by Vanderjagt *et al.* [38]. These results are in keeping with values in adult African -American SCA patients reported in 2003 in which there was a higher than normal reference in plasma triglyceride but lower values of total cholesterol, high density lipoprotein (HDL) and low density lipoprotein (LDL) [39]. The findings are also in keeping with data of Indians in 2016 [40] and Senegalese in 2014 [41]. By implication, hypocholesterolemia, low LDL and HDL and higher values of TG and VLDL reported severally in SCA patients may not be related to age, race, socio-economic status or diet but may be genetic and related to the pathophysiology of the disease. Our study also reported statistically significant higher mean values of the ratios of LDL/HDL, TG/HDL, TC/HDL and AIP in SCA than controls (p < 0.05). TG/HDL ratio was reported higher among the Senegalese SCA patients while AIP was higher in the Indians Sickle cell anaemia patients than controls. The consequence of this is that, SCA patients are more predisposed to developing atherosclerosis and cardiovascular diseases than HbAA controls based on the reported AIP's predictive role to developing coronary heart disease [29].

A study on lipid homeostasis in SCA is necessary because red blood cells membrane is made up approximately 50% of lipid and plasma phospholipids contributes significantly to the synthesis of erythrocytes membranes [42]. Plasma non esterified fatty acids (NEFA) are also building blocks of erythrocyte membranes and its alteration could impact on the structure and function of red blood cells [43]. Therefore, abnormal lipid homeostasis could alter red blood cell membrane fluidity and functions leading to a significant worsening in sickle cell anaemia [44]. Abnormalities in total cholesterol either an increased or decreased level is associated with increased mortality from all causes [45]. Several authors have reported lower than reference value of cholesterol in SCA than general population [46-48]. Though, in our study the mean values of these parameters were generally lower and statistically significant in SCA than in controls, they were within normal reference ranges (Table 2). Similar findings were reported amongst Nigerians in 2017 [49].

It is well established that various haemolytic anaemias with high erythropoietic activity including sickle cell anaemia have been described to be associated with hypocholesterolemia [50]. The pathogenesis of sickle cell anaemia-lipid associated abnormalities have been linked to high erythropoietic activity because of increased cholesterol use, defective liver function secondary to iron overload and malfunctioning post absorptive plasma homeostasis of fatty acids in sickle cell anaemia [51]. The clinical relevance of hypocholesterolemia in SCA include a risk factor to developing depression and suicidal tendencies as reported in 1994 [52]. It may also increase probability of mortality in SCA than in controls [53, 54]. Weather impacts on serum cholesterol and triglycerides. The latter is reported normally lower in winter than in summer, while cholesterol is higher in winter than summer [55, 56]. This study was carried out in rainy season in Nigeria which could have impacted on the results. Prolonged tourniquet application between 2-5 minutes before sample collection which was avoided during samples collection in this study is known to increase cholesterol level from 5 to 15% [57]. Disease conditions such as hypothyroidism and nephrotic syndrome also impact on LDL- cholesterol, VLDL-cholesterol and total cholesterol by increasing their levels [58], while infection and inflammation may decrease total cholesterol and HDL cholesterol and increase triglycerides [59] Dilutional effect due to a postural change from an upright to a supine position could reduce the cholesterol levels by 10% and triglycerides by 12% [60]. All these could have influenced the AIP results obtained for SCA and controls and are all possible limitations of the study.

Our study participants were fasted before blood samples were drawn for lipid profile, should a lipid profile sample be fasting or non-fasting? European and National Cholesterol Education Program (NCEP) guidelines recommend fasting lipid profile samples for cardiovascular risk assessment [61]. Secondly, postprandial triglycerides may be raised several hours after meal [62] and most reference values for lipid profiles are on fasting samples. However, total cholesterol, HDL-cholesterol and non-HDL cholesterol (total cholesterol-HDL cholesterol) remain unaltered in fasting and non-fasting samples [63]. Similarly, little difference exists between values of lipoproteins and apolipoproteins in fasting and non-fasting states which are all associated with a good cardiovascular risk prediction [62]. Bansal *et al.* [64] and Nordestgaard *et al.* [65] independently concluded that non-fasting triglycerides may be a better predictor of cardiovascular risk as compared to fasting triglycerides which is contrary to European and NCEP guidelines. AIP, apart from having stronger sensitivity reflecting interactions between artherogenic and protective lipoproteins thus being a better predictor of coronary artery disease, another advantage it has over single lipid profile is its ability for correction when there is no normal distribution because it is calculated in logarithm, its calculation however, requires no extra cost. Future researches on AIP should focus more on its link with metabolic syndrome, diabetes mellitus [66] and oxidative stress [67].

## Conclusion

AIP value is higher in sickle cell anaemia patients than controls and the former have lower mean values of TC, HDL and LDL than controls and higher mean values of TG and VLDL.

### What is known about this topic

- Patients with sickle cell anaemia have an increased risk of development of cardiovascular disease and venous thromboembolism;
- Dyslipidaemia is the most important risk factor for the development of cardiovascular disease;
- AIP (artherogenic index of plasma) is strongly predictive of cardiovascular disease.

### What this study adds

- Patients with sickle cell anaemia have higher AIP compared with HbAA phenotype control;
- They therefore have an increased risk of development of cardiovascular disease.

## Competing interests

The authors declare no competing interests.
